# Dentists Behavioral Factors Influencing Early Detection of Oral Cancer: Direct Clinical Observational Study

**DOI:** 10.1007/s13187-020-01903-1

**Published:** 2020-10-22

**Authors:** Mohammed Jafer, Rik Crutzen, Esam Halboub, Ibtisam Moafa, Bart van den Borne, Amal Bajonaid, Alhassen Jafer, Ismaeel Hedad

**Affiliations:** 1 Department of Preventive Dental Science, College of Dentistry, Jazan University, Jazan, Saudi Arabia; 2 Department of Health Promotion, Maastricht University/CAPHRI, P.O. Box 616, 6200 MD Maastricht, The Netherlands; 3 Department of Maxillofacial Surgery and Diagnostic Sciences, College of Dentistry, Jazan University, Jazan, Saudi Arabia; 4 Department of Oral Medicine, Infection, and Immunity, Harvard School of Dental Medicine, Harvard University, Cambridge, USA; 5 Dental Division, Ministry of Health, Jazan, Saudi Arabia; 6 Jazan University, Jazan, Saudi Arabia

**Keywords:** Early detection, Oral cancer, Behavior, Clinical practice, Determinants, Oral cancer screening, Patient education

## Abstract

This study aimed to investigate the possible factors affecting dentists’ behavior relating to performing oral cancer examinations as part of routine clinical examination. A total of 95 direct clinical observation sessions—utilizing an instrument consisting of 19 evidence-based observational criteria for oral cancer examinations—were observed by four calibrated dentists. Thirty-two final-year students, 32 interns, and 31 faculty members of Jazan Dental School were examined between April 9 and May 4, 2017. A descriptive analysis was conducted to investigate the frequencies/percentages of the performed observing criteria by all examiners. ANOVA and Tukey tests were carried out to investigate the difference between the examiner groups. A total number of 32 patients participated in the study, whereby each patient was examined by three different examiners from each group, as well as by the attending observer/s. Fewer than 50% of the examiners performed the clinical steps necessary for an oral cancer examination—for example, taking into account past medical history, as well as extra and intra-oral examinations. More than 90% of the examiners examined hard tissue, whereas fewer than 30% of them educated their patients about possible risk factors. A significant difference between examiner groups was found in favor of faculty members. A gap between knowledge and actual practice of oral cancer examinations was evident: majority of participants failed to perform the necessary steps for an oral cancer examination. Previous experience and confidence in performing oral cancer examination are possible explanations for the dentist’s behavior toward oral cancer examination.

## Introduction

Global incidences of oral cancer are still rising, with South Asian countries having the highest incidence rates [1]. In Saudi Arabia, oral cancer—mainly squamous cell carcinoma—is among the most frequently occurring cancers in terms of incidence rates. Most reported cases came from the Jazan region of Saudi Arabia [2, 3]. Several possible factors could put people at a significantly higher risk of contracting oral cancer: using tobacco, in particular the smokeless form of it, and heavy alcohol usage [4–7]. Evidence on systematic disease association with oral cancer is not yet conclusive, except for diabetes, autoimmune diseases, and a few separate syndromes [8–10] .Evidence showed a weak association between dermatological conditions and oral cancer; nevertheless, it commonly manifests itself among patients with dermatological diseases [17]. Furthermore, the risk of oral cancer increases tremendously when a first-degree relative has a history of oral cancer [18, 19].

Most oral cancer cases are detected at a late stage when the tumor has already metastasized to another location in the body [20]. Several studies endorse the fact that the early detection of oral cancer leads to a better prognosis for the disease and better survival rates, and this can be achieved through routine dental clinical examinations [21–24]. An insufficient examination contributes to a delay in the detection of oral cancer, which hampers the prognosis of the disease and greatly affects the 5-year survival rate of oral cancer patients [25]. Factors that might influence dentists’ behavior in terms of practicing routine oral cancer examinations are not fully understood [26]. Possible determinants of the dentists’ behavior can be abstracted individually from a previously published work—for example, dentists’ knowledge [27], awareness [28], perceptions [29], experience [27], limited clinical examination time [30], and focusing on previously examined conditions [31]. Moreover, some dentists reported that they did not perform oral cancer examinations because they were worried about their patients’ reactions toward oral cancer examinations [32]. Therefore, the present study investigates possible explanations for dentists’ behavior by directly observing routine clinical dental examination sessions.

## Methods

The present study was performed according to the ethical standards of the institutional research committee, as well as the 1964 Helsinki Declaration. It received ethical approval from Jazan University (registry no. [CDREC-06]), dated 21 December 2016. All participants have consented prior to their participation, including with regard to the publication of the findings. The reporting of this present study followed the STROBE guidelines for reporting cross-sectional studies [33].

The present study utilized a descriptive cross-sectional study design, in which direct clinical observation was carried out among final-year dental students, interns, and faculty members between April 9, 2017, and May 4, 2017. The main targets of this study were the dental interns who are the first line of treatment in JDS clinics and who oversee the completion of the primary dental charts of all new patients. The total number of interns was 40 at the time of planning this study. A personal invitation was sent to all the interns and an explanation of the study process was delivered to them in the form of two discussion sessions by the principal investigator (PI). To minimize effects on the behavior of clinicians under observation in dental examination sessions, the study and its process were explained to the participants without mentioning that the study would focus on the dental examinations and, in particular, oral cancer screening performance and the required related steps as a follow-up. We aimed to reduce the Hawthorne effect [34] (i.e., when the observer influences the examiners’ behavior) by notifying the observers to remain unreactive while observing the sessions and to remain passive. All 32 interns who accepted the invitation to participate were informed later of their scheduled appointment to examine a patient in a designated clinic that was booked for the study. Afterwards, final-year dental students and Arabic-speaking faculty members were invited to participate in the study, and later those who accepted received an explanation of the study and related aspects in the same aforementioned process for interns. Four faculty members (dentists) from JDS were the observers in the dental examination sessions, three of whom are also authors of this study, with one acting as the main observer. The rule for the observer was to observe the examiner’s practice and to subsequently mark the performed action in regard to the checklist items.

To ensure the study was conducted as a form of routine dental examination at JDS clinics, it was decided that all the study’s clinical examinations would be conducted on new dental patients from the waiting list of JDS clinics. To compare between the three different groups of clinicians, dental students, interns, and faculty members, it was decided that each patient would be examined by a dental student, intern, and a faculty member, as well as an observer. Therefore, to reach the target number of 32 new patients who are willing to participate and be examined by three clinicians and available observer/s, around 93 patients were contacted and invited over the phone by the principal investigator from the JDS clinics’ patient relation office. This process was repeated later to invite nine more new patients to replace the five patients who did not show up to their appointment and four patients who refused to be examined by all three examiners.

An instrument for the observation of oral cancer screening practice in form of checklist was developed and was based on the current available evidence relating to the recommended practice of oral cancer screening. The observation instrument included the most appropriate clinical steps to be taken during comprehensive clinical examinations of dental patients [35, 36], as well as the risk factors of oral cancer [37–39]. At first, the instrument consisted of 32 items in the form of a checklist, with two labeled boxes for the options “done” and “not done.” However, 15 items (11 to 25) were condensed to 6 items (11 to 16) after reaching a consensus from four oral cancer consultants, who evaluated the proposed observational instrument, to be able to fully reflect on the examiners’ performance of the targeted screening steps. Both checklists are available at <https://osf.io/4v9gr/>. Therefore, the utilized observational instrument in the present study finally consisted of a 19-item checklist, with the first 17 items relating to the essential steps of routine oral cancer screening, and the last two items were for when further diagnostic/evaluation step(s) were required. Items 1–10 related to reporting the patient’s general health, family history of cancer, habits, and diet. Items 11–16 related to the performance by the examiner of different steps for the head, neck, and oral examination. Item 17 was for obtaining a plain radiograph, and items 18 and 19 were for conditions when the further evaluation was necessary to diagnose potential positive oral cancer cases. Each item was given a weight of one, two, or three, based on the degree of significance and relevance as supported by current evidence [4–19, 39–53], with one standing for items of the least significance and three for items of the most significance. (Please see Table 1 for more detailed information on each of the items with the designated weight and supportive evidence.) The sum of the performed item weights from the checklist was used as the main outcome of the study.

**Table 1:** Justification table for the selected items in the instrument

No.	Observing criteria	Weight	Justification	Reference
1	Systemic diseases	1	Evidence on systematic disease association with oral cancer is not yet conclusive, except for diabetes, autoimmune diseases and a few syndromes	[8–10]
2	Infectious Diseases (HPV, HIV, HBC, etc.)	1	Their association with oral cancer is a foregone conclusion. It is given a weighting score of one, as these infectious diseases are less prevalent in the Jazan region	[11–16]
3	Dermatologic conditions	1	Evidence showed weak association with oral cancer but is a common manifestation among patients with dermatological diseases	[17]
4	Medication (immunosuppressive, anti-inflammatory antihypertensive, and steroids delivered in inhaler/ topical/oral form)	1	Evidence on its association with oral cancer is scarce	[42]
5	Previous family cancer history (type and associated treatment)	1	The risk of oral cancer is increased tremendously when a first-degree relative has a history of oral cancer	[18, 19]
6	Tobacco smoking (frequency and duration)	3	Smoking Tobacco, Smokeless Tobacco, and drinking Alcohol are well-known major risk factors for oral cancer. However, Alcohol was given two as it is illegal in Saudi Arabia and is not common in the region	[24–27]
7	Smokeless tobacco (habit type, frequency, and duration)	3		
8	Alcohol (frequency and duration)	3		
9	Diet (antioxidant, minerals, etc.)	2	Although a substantial body of evidence demonstrated its role in preventing oral cancer e.g. vitamin A (retinol), E (α-tocopherol); and carotenoids (β-carotene), diet is a loose term and cannot be retrieved very well while reporting patient history	[6, 28–31]
10	Oral hygiene (heavy bacterial load, acetaldehyde production)	1	Few studies reported an increased risk of oral cancer with poor oral health. However, these studies carry many confounding factors that affect its strength	[33–36]
11	Palpate for enlarged lymph nodes of *the neck*	3	Almost all oral squamous cell carcinomas are preceded by visible mucosal changes, such as white, red, or mixed patches, lymph nodes tenderness, palpation of abnormal mucosal findings on the lip; cheek;lateral, dorsal and ventral surfaces of the tongue; palate; floor of the mouth and teeth and their supporting structures are essential steps in oral cancer screening. These steps facilitate early detection of the disease and improve the treatment and survival rate. Unfortunately, these structures are skipped during head and neck examinations by most of dental practitioners	[37–39, 42]
12	Examining *upper and lower lip mucosa* and *buccal mucosa*	3		
13	Examining the lateral and ventral of the *tongue* (white and red patches) and *tongue* lumps (feeling the tongue lumps)	3		
14	Examining the *palate*	3		
15	Examining the *floor of the mouth*	3		
16	Examining *dentitions* and supportive structure	3		
17	Obtaining plain *radiographs*	1	Obtaining a radiograph is given a weighting of one as it is not recommended by the previous studies in the context of epithelial tumors. However, it may be useful in demonstrating the extent of cortical bone invasion in large tumors	[43, 44]
*18	Additional *diagnostic tests* relevant to the evaluation (biopsy, other devices for diagnosis) and consulting specialist/s	3	A biopsy is a must for any abnormality with features of potential malignancy or when it does not respond to two-week treatment protocols. Referring a suspicious case to a specialist for further evaluation and confirmation is a must, as well. As these two items are not done routinely, they are used as extra items in case the initial screening reveals (a) suspicious lesion(s)	[40, 41]
*19	Advice/s on oral cancer risk factors	3		

*If needed

Prior to the initiation of the present study, a thorough discussion session including observers and the PI was conducted to acquaint all with the observational instrument and the logistics of the study. Afterwards, two hands-on clinical training sessions were provided with two volunteers, who acted as the patient and the examiner to facilitate the training sessions. This was also to provide the observers with experience in handling the checklist while also carefully observing the examiner performing the clinical examination as well as patients’ responses. A feedback discussion session for observers, which included the PI, was conducted after the two training sessions to reflect on the experience, as well as to discuss and reach a consensus regarding the appropriate approach for handling the observational sessions and checklist. Then, for the internal validity testing of the observational instrument, eight dental examination sessions—which included eight volunteer patients and eight volunteer dentists—were overseen by all observers, whereby interrater reliability was investigated using an interclass correlation coefficient test that showed significant consistency (*p* < .000). Prior to the present study’s dental examination sessions, each participating patient was first examined by the observer/s who then observed the clinicians’ dental examination sessions. All 95 dental examination sessions were observed by the main observer and mentored by the PI. In addition, 18 of these sessions were observed by all the observers, while others were overseen by two or three observers (see Table 4).

Descriptive analyses were run to investigate the participants’ demographics, as well as the frequency of the individual performance items in the observational checklist by the main observer. Furthermore, paired *t* tests and correlation tests were run to investigate any relationships between the main observer’s findings and other observers’ findings. Later, using total score by the main observer and mean total score by all observers, independent *t* tests were run to investigate any differences between examiners’ genders as well as patients’ gender. Similarly, ANOVA tests were run to investigate any differences between examiners’ occupations (final-year dental students, interns, and faculty members) with Tukey’s HDS comparison between each occupation category. Additionally, Pearson’s correlations were run to investigate any correlation between patient age and total score by the main observer and mean total score by all observers. A qualitative approach was utilized to perform post-exam investigations in the form of two focus group discussions (FGDs) with the participants of the current observation study. The FGDs aimed to explore the possible factors explaining the observed findings. The 95 participants in the cross-sectional study were invited to participate in the follow-up discussions by direct contact.

## Results

The total number of participating examiners was 95, with an almost equal distribution of gender and occupation (final-year dental students, interns, and faculty members). The total number of participating patients was 32, with ages ranging between 19 and 70 years old and with 62.5% males (see Table 2).

**Table 2:** Demographics of Participants

	*n	Frequency	%	*M*	SD
Examiner	95				
Sex					
Male		48	50.5		
Student		16	33.3		
Intern		16	33.3		
Faculty member		16	33.3		
Female		47	49.5		
Student		16	34		
Intern		16	34		
Faculty member		15	31.9		
Patients	** 32				
Age				38.6	14.4
				(Range: 19–70)	
Sex					
Male		20	62.5		
Female		12	37.5		

*Number of participating examiners, as observed by the main observer

**Number of participating patients; however, 95 was the total number of clinical dental examinations, as observed by the main observer

Table 3 shows the frequency of the observed items by the main observer; 70% of examiners investigated the systemic diseases of their patients, while fewer than 30% of examiners investigated their patients’ tobacco use and their oral hygiene practices. Moreover, 90% of the examiners checked their patients’ dentations, while fewer than 50% of them examined their patients for enlarged lymph nodes of the neck, lip, check, tongue, palate, and floor of the mouth. Furthermore, among the participating patients, three had suspected lesions that required further investigation, and 14 required advice regarding oral cancer risk factors, according to the main observer. However, among participating examiners, only three female final-year dental students out of nine examiners had requested specialist consultations, as well as only 11 examiners out of 42 providing advice to the patients who needed it (see Table 3).

**Table 3:** Descriptive findings

**W 43	No.	***Observing items	Frequency of performed item							Total in %
			Total	Student		Intern		Faculty		
				M/16	F/16	M/16	F/16	M/16	F/15	
1	1	Systemic diseases	68	8	12	12	12	11	13	71.6
1	2	Infectious diseases (HPV, HIV, HBC, etc.)	8	0	1	0	2	5	0	8.4
1	3	Dermatologic conditions	2	0	0	0	0	1	1	2.1
1	4	Medication (immunosuppressive, anti-inflammatory antihypertensive, and steroids delivered in inhaler/ topical/oral form)	34	4	6	5	6	6	7	35.8
3	5	Previous family cancer history (type and associated treatment)	5	1	2	0	0	1	1	5.3
3	6	Tobacco smoking (frequency and duration)	18	2	4	1	3	5	3	18.9
3	7	Smokeless tobacco (habit type, frequency, and duration)	26	4	8	1	3	8	2	27.4
2	8	Alcohol (frequency and duration)	2	0	0	0	0	2	0	2.1
2	9	Diet (antioxidant, minerals, etc.)	7	0	1	0	1	4	1	7.4
1	10	Oral hygiene (heavy bacterial load, acetaldehyde production)	18	5	1	0	2	6	4	18.9
3	11	Palpate for enlarged lymph nodes of the *neck*	32	7	8	0	4	7	6	33.7
3	12	Examining *lips* and *cheek*	46	10	8	5	5	9	9	48.4
3	13	Examining the sides and underside of the *tongue* (white and red patches) and *tongue* lumps (Feeling the tongue lumps)	41	8	8	5	6	4	10	43.2
3	14	Examining the *palate*	14	1	2	2	1	4	4	14.7
3	15	Examining the *floor of the mouth*	22	2	3	4	1	6	6	23.2
3	16	Examining *dentitions* and supportive structure	86	15	13	14	14	16	14	90.5
1	17	Obtaining radiographs	20	3	3	5	5	2	2	21.1
3	*18	Additional *diagnostic tests* relevant to the evaluation (biopsy, other devices for diagnosis) and consulting specialist/s if needed	3/9	0	3	0	0	0	0	33.3
3	*19	Advice on oral cancer risk factors if needed	11/42	0	6	0	1	3	1	26.2

*If needed

**Items weight

***As completed by the main observer

There were statistically significant correlations between the main observer and other observers, with the strongest correlation (*r* = .807) between the main observer and the second observer (see Table 4). Moreover, no statistically significant differences were found between the main observer and other observers (second observer *p* = .571 and fourth observer *p* = .062), except for the third observer (*p* = .018); see Table 4. Furthermore, there were no statistically significant differences between all 95 examiners based on their sex. However, there was a statistically significant difference based on the examiners’ occupation (see Table 5). The statistically significant difference was found between faculty members and interns, using both the total score by the main observer and the total mean score by all observers (*p* = .007 and *p* = .031, respectively) (see Table 6). On the other hand, there were no statistically significant differences found between examiners based on the patients’ age or sex (see Table 7).

**Table 4:** Paired *t* tests and correlation tests between observers (*N* = 95*)

	2nd observer	3rd observer	4th observer
Main observer	*n* = 29	*n* = 38	*n* = 18
	1 *M* = 34.9, SD = 21	1 *M* = 34.2, SD = 20.3	1 *M* = 33.5, SD = 22
	3 *M* = 36.3, SD = 20.4	2 *M* = 28, SD = 15	4 *M* = 25.2, SD = 13
	*M* = − 1.4, SD = 12.9	*M* = 6.3, SD = 15.5	*M* = 8.3, SD = 17.6
	95% CI for difference [− 6.28–3.53]	95% CI for difference [1.14–11.36]	95% CI for difference [− 0.48–17.07]
	*t* (28) = − 0.574	*t* (37) = 2.48	*t* (17) = 1.994
	*p* = .571	*p* = .018	*p* = .062
	*d* = 0.034	*d* = 0.175	*d* = 0.156
	*r* = .807	*r* = .648	*r* = .584
	p = .000	*p* = .000	*p* = .011

*Number of clinical dental examination sessions, as observed by the main observer

**Table 5:** Independent *t* tests and ANOVA tests for examiners (*N* = 95*)

	* Sex	° Sex	* Occupation	° Occupation
Total score	♂*M* = 29.8, SD = 20.3	♂*M* = 28.2, SD = 17.2	*F* = 4.944 *p* = .009 *η*_p_^2^ = 0.097	*F* = 4.081 *p* = .020 *η*_p_^2^ = 0.081
	♀*M* = 30, SD = 14.8	♀*M* = 29.8, SD = 14		
	95% CI for difference [− 7.47–7.03]	95% CI for difference [− 8.02–4.74]		
	*t* (86) = − 0.061	*t* (93) = − 0.510		
	*p* = .952	*p* = .611		
	*d* = 0.005	*d* = 0.051		

*Number of participated examiners as observed by the main observer

°Using mean total scores by all observers

**Table 6:** ANOVA comparisons of examiners (*N* = 95*)

Group		Mean difference	Tukey’s HSD comparisons (*p* values)		95% confidence interval
			Student	Intern	
Intern	Main observer	− 8.48	.120		− 18.63–1.65
	All observers	− 8.88	.054		− 17.88–0.11
Faculty member	Main observer	4.82	.502		− 5.40–15.04
	All observers	0.91	.968		− 8.15–9.98
Faculty member	Main observer	13.31		*.007*	3.08–23.53
	All observers	9.80		*.031*	0.73–18.87

^#^*p* value *< .05*

**Table 7:** Independent *t* tests and Person correlation tests for examiners based on patients (P) (*N* = 95*)

	* P sex	° P sex	* P age	° P age
Total score	♂*M* = 31.5, SD = 17.7	♂*M* = 30.1, SD = 14.3	*r* = .076, *p* = .461	*r* = .062, *p* = .551
	♀*M* = 27.3, SD = 17.5	♀*M* = 27.2, SD = 17.5		
	95% CI for difference [− 3.28–11.59]	95% CI for difference [− 3.66–9.45]		
	*t* (93) = 1.110	*t* (93) = 0.877		
	*p* = .270	*p* = .383		
	*d* = 0.119	*d* = 0.091		

*The number of participating patients was 32; however, 95 was the total number of clinical dental examinations, as observed by the main observer

°Using mean total scores by all observers

A total number of 23 participants have accepted to participate in the two follow-up FGDs. The two FGDs revealed a number of factors that are possibly associated with the observed items’ scores. The majority of the 23 participants stated that the presence of questions related to items 1, 4, 12, and 16 in the JDS clinical charts made them used to do it. Dependence on previous dental examination is a factor that was elicited by the majority of the participants to be generally related to the low-score items in the checklist. Another factor that reached agreement by all participants is the lack of confidence to identify oral precancerous/cancerous lesion, to provide tailored risk factor education or to provide tobacco counseling as they lacked formal training on these skills.

Majority of participants thought it is not important to ask patients about items 3 and 9 (dermatologic conditions and diet) as they assumed it has no clear relationship with their patients’ oral health. Participants linked the cultural and religious unacceptability of alcohol use to the observed low score in item 8 (alcohol). For items 6, 7, and 19 (tobacco smoking, smokeless tobacco, and advice on oral cancer risk factors), female students and interns had higher scores than their counterpart. The participants mentioned that female students/interns are more vigilant to the oral changes associated with tobacco as they are used to examine mainly female patients who are usually non-smokers. Moreover, female participants mentioned that the tobacco advice they had given to the patients was based on their personal beliefs, as they did not receive formal training on tobacco counseling. In relation to item 17 (obtaining radiograph), participants denoted the reason for obtaining radiograph is related to the chief complaint only. Dental interns revealed that two factors could be related to their general low score in comparison to students and faculty members. The first one was because they rely on the other dentists whom the patient will be referred to do the next oral care/treatment. While the second factor was because they cannot perform full oral screening on each patient as they have a busy clinical schedule with large number of patients.

## Discussion

The present study investigated possible explanations for dentists’ behavior by means of direct observations of routine dental clinical examination sessions. The interns in this study were recruited from the same group that were evaluated in two recent studies that included students, interns, and faculty members for their knowledge, attitude, self-efficacy, and opinions regarding oral cancer practice [32, 54], in which oral cancer knowledge of dental interns was found to be adequate [54]. In addition, favorable perceptions among dentists toward oral cancer practices were found regardless of the reaction of patients [32]. However, the experience and the confidence to perform oral cancer examinations and educate patients on risk factors was found to be limited to dentists specialized in fields related to oral cancer [32]. The factors relating to time constraints and previously examined conditions were controlled for in this study, as no time restriction was placed on dental examination sessions and all were carried on new patients. The overall findings of the study indicated that the examiners performed the clinical steps with which they had more experience and higher confidence, in terms of performing and understanding the potential treatment modalities and outcomes. The majority of them examined the dentations of their patients and asked for patients’ systemic diseases, which could be necessary for their usual practice. However, fewer than half of the examiners performed other extra- and intra-oral examination steps and less than one-third of them investigated their patients’ potential risk factors for oral cancer. This is also supported by the statistically significant difference favoring the faculty members’ group, as they have a higher level of experience and are confident in performing oral cancer examinations.

The knowledge of oral cancer among dentists has been investigated thoroughly in previous studies and postulated to be related to dentists’ practice of oral cancer examinations [27–29, 55]. However, knowledge (or lack thereof) alone is not enough to explain, for example, why dentists are not performing oral cancer examinations in their routine practice. According to behavior-change theories, such as the Reasoned Action Approach, the Social Cognitive Theory and the I-Change Model, knowledge only has a distal influence on the individual’s behavior and its effect is often limited when trying to explain complex behaviors; such as in our case: dentists’ practice of oral cancer examinations [56–58]. This is in line with the findings of this study, as most participants failed to perform oral cancer examinations even though they had adequate knowledge of oral cancer [54, 59, 60]. This observation has highlighted the important role of dentists having actual control over their practice of oral cancer examination.

Dentists have actual control over their practice of oral cancer examinations when they demonstrate their ability to perform the following essential sub-behaviors: extra- and intra-oral screening skills, obtaining radiographs, taking a biopsy, writing referral reports, specialist consultations, communicating with or counseling patients (e.g., smokeless tobacco users), specifying an oral cancer provisional treatment plan (treatment modalities, outcome), and referring suspicious cases to specialized centers (Table 1). Additionally, the lack of these basic sub-behaviors can adversely affect dentists’ practice of oral cancer examination, either directly or indirectly via self-efficacy. As described in the Social Cognitive Theory by Albert Bandura, an individual’s confidence in his capability to produce the desired effect through his actions constitutes a core belief that operates via its impacts on various processes—both cognitive and affective [57]. Among the four main sources that enhance self-efficacy, the individual’s experience (mastery and vicarious/modeling) is recognized as creating and strengthening a stronger sense of self-efficacy. This suggests that a dentist’s efficacy can be influenced by his own experience in practicing oral cancer examinations. In addition, observing other dentists succeeding at practicing oral cancer examinations is able to reinforce the observing dentist’s belief in his own capability to master comparable actions required to succeed. Hence, dentists’ practice of oral cancer examinations could be greatly influenced by their previous experience and confidence in their ability to perform oral cancer examinations [56, 57].

Direct clinical observation methods strengthen this study by capturing the clinical steps that dentists may or may not have performed, which leads to a better understanding of the behavior’s potential causes. Due to the cross-sectional nature of this study, the findings need to be tested through experimental study design, in order to measure the effect of experience, skills, and self-efficacy on dentists’ behavior. Furthermore, having a main observer present, who observed all dental clinical examination sessions, added to the reliability of the comparison between different examiners. Similarly, having four observers to compare findings with added to the internal validity of this study. Moreover, the observational instrument that was developed for the study had not been tested in previous independent work. However, the findings of this study indicated that the developed instrument had the capacity to investigate oral cancer examinations as part of routine dental clinical examinations. All observers had strong statistical correlations, with no statistical difference being found between the three of them.

To conclude, the present study has shed light on the gap that existed between knowledge and actual practice of oral cancer examinations by dentists. The practice of oral cancer examinations is a complex behavior that is influenced by multiple factors: oral cancer knowledge, perceptions, experience, self-efficacy, actual control, and other external factors such as the afforded clinic time per patient. Furthermore, experience and confidence are essential determinants for performing oral cancer examinations. Therefore, dental schools and decision-makers should be aware about the influence of these determinants on oral cancer examinations and should be stressed in the future interventions that intend to improve the practice of oral cancer examinations as part of routine dental clinical examination sessions.
